# The Role of Attentional Control in Attention to Emotional Stimuli Among Middle-Aged and Older Adults

**DOI:** 10.1093/geroni/igaa057.1624

**Published:** 2020-12-16

**Authors:** Yuen Wan Ho, Derek Isaacowitz

**Affiliations:** Northeastern University, Boston, Massachusetts, United States

## Abstract

Prior studies have examined age differences in attention to emotional stimuli; in the current study, we considered how this might relate to dispositional measures of attentional control across age groups. Participants were 116 middle-aged (aged 35 – 64 years) and 39 older (aged 65-86 years) adults in the United States. In the study, participants filled in the Emotional Attentional Control Scale. Then participants watched fearful, happy, neutral, and disgusting videos. The gaze time for each video was measured by an eye tracker. Results did not show significant age difference in attention to happy and neutral videos. However, middle-aged adults gazed relatively more to the disgusting video and relatively less to the fearful video, t (115) =2.16, p =.03. The opposite pattern was found among older adults, t (38) =5.85, p<.001. Self-reported emotional attentional control was not significantly related to attention in either age group. These findings suggest that different stimuli may yield age differences in fixation that are a less consistent pattern with the age-related positivity effect reported in previous studies, and also that self-reported emotional attentional control may not relate to gaze patterns.

